# The Challenges of Prolonged Gas Sensing in the Modern Urban Environment

**DOI:** 10.3390/s20185189

**Published:** 2020-09-11

**Authors:** Shai Kendler, Asaf Zuck

**Affiliations:** 1 Environmental Science Division, Israel Institute for Biological Research 24 Lerer St., Ness Ziona 74100, Israel; asafz@iibr.gov.il; 2 Department of Environmental, Water and Agricultural Engineering, Faculty of Civil & Environmental Engineering, Technion—Israel Institute of Technology 616, Rabin Hall, Haifa 32000, Israel

**Keywords:** air pollution, urban environment sensing, aerosol, dust, sensor durability, cyclone dust separator

## Abstract

The increase in the urban population is impacting the environment in several ways, including air pollution due to emissions from automobiles and industry. The reduction of air pollution requires reliable and detailed information regarding air pollution levels. Broad deployment of sensors can provide such information that, in turn, can be used for the establishment of mitigating and regulating acts. However, a prerequisite of such a deployment strategy is using highly durable sensors. The sensors must be able to operate for long periods of time under severe conditions such as high humidity, solar radiation, and dust. In recent years, there has been an ongoing effort to ruggedize sensors for industrial applications with an emphasis on elevated temperature, humidity, and pressure. Some of these developments are adapted for urban air sensing applications. However, protection from dust is based on filters that have not been modified in the last few decades. Such filters clog over time, thus requiring frequent replacement. This editorial presents the need for a consumable-free dust removal device that provides consistent performance without affecting the sensing process. A specific solution for removing dust using a cyclone dust separator is presented. The cyclone dust separator is implemented as an add-on module to protect commercially available sensors.

## 1. Introduction

Urbanization processes result in many cases in the deterioration of air quality due to the increased emission of organic vapors, pollutant gases, and combustion products, including aerosol emissions from anthropogenic sources such as automobiles and industry [1,2,3]. Reducing air pollution requires considerable efforts to improve urban infrastructures and to regulate emissions. Such actions require prolonged air quality monitoring to provide the required information regarding the concentration of the pollution and its source (location and mass-flow rate) [4,5,6,7]. This paper describes the technological challenge of prolonged gas sensing in the urban environment. Such a mode of operation exposes the sensors to solar radiation, non-optimal temperatures, and humidity and aerosols that may degrade its performance. In many cases, the sensor can be ruggedized by utilizing technologies that are common in industrial applications. However, technological solutions to minimize the adverse effects of aerosol on gas sensors have not been improved in the last few decades. This paper describes a new alternative approach that may address this gap based on cyclone dust separators that can be used as an add-on unit to gas sensors without modifying them.

Traditionally, air quality monitoring is performed using stationary stations equipped with a small number of sensors to detect the concentrations of gases such as carbon monoxide, nitrogen oxides, ozone (“gases”), and particulate matter. These sensors are large, expensive, and designed for “fixed-site” installation [8]; hence, vast deployment is impractical. In recent years, there has been considerable effort to miniaturize low-cost sensors that can be deployed in large numbers or mounted on a moving platform to provide detailed mapping of the pollution concentration. For example, Rohi et al. developed a drone-based miniature gas sensor array for several polluting gases and particulate matter [9]. Such a highly portable system can provide detailed mapping of the pollutant concentrations. However, the complexity involved in the continuous operation of a drone fleet and the limited flight time is a technological barrier that should be considered. Brienza et al. developed a miniaturized ground sensor (“uSense”) that can be deployed as part of Wireless Distributed Sensor Networks (WDSN) to monitor air quality in an urban area. This low-cost and low-power sensor can potentially be deployed in large numbers, creating a high-resolution grid, and provide detailed information both in space and time regarding the concentrations of several polluting gases. The uSense was tested in urban areas, and the obtained gas concentrations coincide with those reported by the authorities using standard stationary stations.

Accurately measuring the gas concentration requires meticulous sensor calibration [10]. Moltchanov et al. deployed a network of miniature wireless multi-sensors in several urban sites and proposed an on-site calibration process to obtain a reliable readout from the sensor even during prolonged operation. Somov et al. also noted that prolonged sensor operation results in changes in the sensor’s response, and periodical calibration is required for such applications [4]. Kizel et al. noted that, after being deployed, sensors’ responses tend to drift over time, and frequent calibration may be required. Since standard calibration techniques are cumbersome, they developed an alternative approach in which only one sensor is calibrated using a calibration gas mixture. The rest of the sensors are calibrated sequentially against the reference detector [11].

The sensors’ calibration drift—and, sometimes, total failure—is a result of various factors that depend on the sensor’s and installation site’s characteristics. Intense solar radiation, elevated temperatures, humidity, corrosive gasses, and dust may result in the sensor’s continuous deterioration and malfunction. Recently, the effect of high concentrations of dust on chemical detectors has been reported [12,13]. Dust accumulation on the optical module of a handheld flame photometric detector resulted in a decrease in sensitivity and eventually in total failure. The rate of this effect depends both on the dust concentration and the operation time [12,13]. One cannot underestimate aerosols’ influence on the performance of the detector during prolonged operation, even in what may be considered convenient locations. Over time, aerosols may settle in the sensor and degrade its performance. This situation is even more challenging in arid locations, which are characterized by a harsh environment, including high dust concentration. For example, Founda et al. found that the air quality in the urban area of Athens deteriorated in the last century due, among other reasons, to an increase in particulate matter concentration according to the observed visibility ranges records since the beginning of the 20th century. These days, events of low visibility ranges are more frequent than ever due to climate change and may occur in other locations, making the impact of aerosols more pronounced.

Several groups have developed ruggedized sensors for different applications. Chen et al. reviewed the development trends of wireless passive LC resonant sensors for harsh environments. In these sensors, the gas affects the sensor’s capacitance (“C”), which, in turn, affects the sensor’s resonance frequency. These changes are detected by another inductively coupled coil (having a typical inductance “L”, hence “LC”) using a dedicated electronic circuit [14]. These sensors are very rugged since they comprise a passive sensor element controlled by a remote antenna placed at more favorable conditions [15]. Ong et al. developed and studied several systems based on this technology. For example, the water content in various matrices such as sand and concrete was measured by placing a passive planar inductor-capacitor (LC) circuit in the interrogated matrix’s material. An antenna tracks the changes in the sensor’s resonant frequency, which is a function of the water content inside the sample. Such a measurement provides information regarding the sample while separating between the robust sensor unit and the electronics [16]. This technology has proven suitable also for gas measurements in a harsh industrial environment when depositing a multiwall carbon nanotube/silicon dioxide (SiO_2_) composite layer on a planar inductor-capacitor resonant circuit sensing element. Adsorption of polluting gases on the sensing element altered its permittivity and conductivity as monitored by an antenna placed outside the test chamber. Interestingly, the adsorption of CO_2_ and O_2_ was utterly reversible, while the adsorption of ammonia partially resulted in non-reversible changes in the sensing element’s permittivity and conductivity [17]. The irreversible adsorption of ammonia on the sensing element’s surface is an example of the potential permanent damage to sensors by corrosive gases.

Automotive emissions are a significant source of air pollution in highly populated areas. Sensing these gases as close as possible to their source provides valuable information but exposes them to a harsh environment of extreme temperatures and pressure changes for a prolonged time [5]. Hence, sensing such emissions requires separation between the sensing element and the sensitive electronic components. Evanescent wave absorption spectroscopy configuration using fiber optics might be a good solution in this case since gas sensing at elevated temperatures as high as 1000 °C has been demonstrated by Ohodnicki et al. [18]. Another example of a sensor that can be adapted for operation in a harsh environment is an “all optical” photoacoustic sensor. A gas sample is irradiated with a tunable light source, and a portion of the light is absorbed by the gas, resulting in pressure changes which are proportional to the gas concentration. Scanning the light source’s wavelengths enables the identification of the gas according to its unique spectral signature. Considerable effort has been devoted to the development of a sensitive optical pressure transducer, resulting in a durable sensor [19].

This paper aims to increase the awareness of the negative impact of prolonged operation of sensors in urban areas on their performance, highlighting the negative effect of aerosols that may accumulate in the sensor and degrade its performance. Today, the standard approach to overcoming this problem is to protect the sensor with particulate filters. Supplying enough energy to draw air through the filter and periodical replacement of clogged filters may provide an expensive yet viable solution in some cases. However, filters have an additional drawback resulting from the adsorption of semi- and non-volatile organic compound vapors in the filter. Such absorption decreases the vapor concentration in the sampled air, resulting in lower sensitivity and longer response and clear-down time. In many cases, it may be possible to extract vapors from the filter by heating it to a sufficiently high temperature [20]. However, this process requires a significant amount of energy, maybe insufficient in some cases, and the heated air itself may affect the sensor performance. An alternative and superior approach based on a cyclone dust separator designed as an “add-on” unit is presented here. Unlike filters, the cyclone is a consumable and practically maintenance-free device that does not adsorb organic vapor; hence, it does not affect the sensor’s performance while providing prolonged operation.

## 2. Specific Challenge—Gas Monitoring in a Dusty Environment

Zipser et al. developed an acoustic sensor capable of in situ measurement of binary gas mixtures in the hot, humid, and contaminated exhaust air of industrial driers [21]. The authors indicated that dust might accumulate on the sensor and negatively affect its operation. The solution, in this case, is using a particulate filter. While particulate filters are handy, they have inherent resistance to airflow, which increases as particles are trapped by the filter, thus increasing energy consumption. Periodically replacing the filters is possible, but one must also consider consumables and labor costs involved in such an operation. Laminger et al. suggested an interesting solution by regenerating filters instead of replacing them [22]. Filters are regenerated by reversing the airflow so that the dust particles are pushed back into the atmosphere. This process requires a dedicated pneumatic module, interrupts detection, and increases energy consumption. Tartakovsky et al. studied ways to regenerate diesel particulate filters by catalytically oxidating soot particles. Note that such a process requires considerable resources, such as catalysts and energy [23].

**Figure 1:**
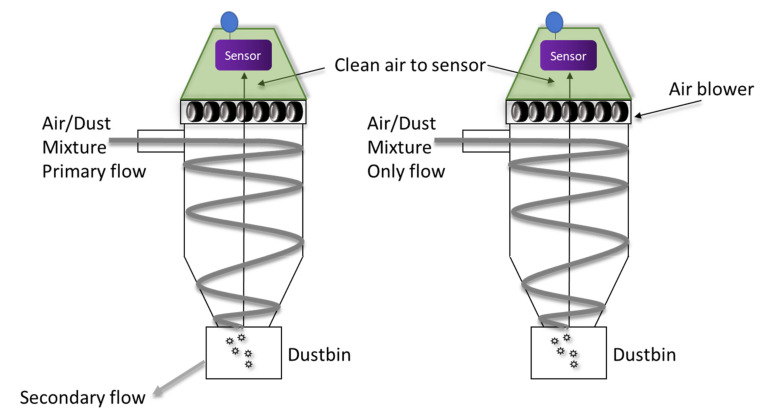
A scheme (not in scale) depicting a cut through the CSR. (**Right**)—a conventional CSR with a single air stream. Air is entering the CSR tangentially, forcing the flow into a spiral path. The centrifugal force moves the dust particles toward the wall of the cyclone where they collide and lose their kinetic energy, then finally fall to a dustbin. Air and vapor exit the CSR and enter the gas sensor (not shown). (**Left**)—an improved CSR. Improvement is achieved by splitting the airflow between the primary flow (typically 85% of the total flow) and secondary flow. Both schemes show a sensor (purple rectangle) coupled to the exit of the CSR. The sensor samples gases and vapors from the air after the dust has been removed and wirelessly transmits the information.

The drawbacks of filters led to the development of an alternative sensor protection approach based on cyclone dust separation (CSR). The CSR is designed to be an add-on unit to a chemical warfare agent detector (CWA) [12,13]. Note that this is a highly demanding application since these sensors must respond rapidly and accurately to extremely low concentrations of the highly toxic CWA, having boiling points between 140 and 300 °C. CSRs are common in industrial applications that require particulate matter removal from a large volume of air with low operational (energy and maintenance) costs. Over the years, many CSR models have been developed, all based on the universal principle of using centrifugal forces to separate particles from the air [24]. A mixture of air and dust enters the CSR tangentially, forcing the flow into a spiral path. The circular flow results in a centrifugal force that moves the dust particles toward the cyclone wall, where they collide and lose their kinetic energy and then finally fall into a dustbin. Figure 1 presents a scheme of the classic and the improved CSR (see text below). The sensor is mounted at the exit of the CSR and allowed to sample the dust-free air. The specific operational parameters and coupling between the CSR and the sensor may be adjusted according to the sensor’s properties and the specific application. The simple fact that the particles are moved away from the gas stream results in consistent performance with no effect on the analyte’s sensor response. Unlike filters, the operation of the CSR is practically maintenance-free. Testing the CSR as an add-on unit to a field-portable CWA detector demonstrated that, when using the CSR, the detector response to CWA vapor is unaffected by the dust particles. At an extremely high dust concentration (0.9 gr/m^3^) which is typical of desert storms, resulting in zero visibility, the sensor performed flawlessly for more than two hours. The CSR described in [12,13] increased the time between failure by approximately two orders of magnitude compared to unprotected sensors in the same conditions. One may argue that the CSR is not a “maintenance-free” device since the dustbin accumulates dust and has to be cleaned periodically. However, if one considers that a CSR operated at a flow rate of 100 L/min constantly faces a dust concentration of 500 micrograms/m^3^, which is three times higher than the average PM10 level in Anshan, China, [25], then the mass of dust collected after one year is ~26 gr. Such an amount will require frequent filter maintenance but is hardly a challenge for a CSR device. 

**Figure 2:**
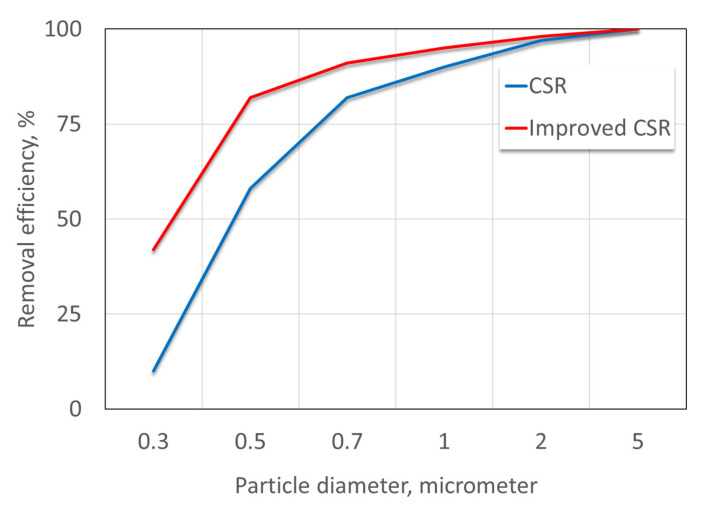
Dust particle removal efficiency as a function of the particle diameter using the conventional CSR (blue line) and the improved CSR (red line). In both cases, the total flow rate is 100 L/min.

The main drawback of the CSR technology is that its efficiency decreases with the particle size. Note that the rate of deposition of fine particles (mass per time unit) in the sensor may be slow due to their low mass and tendency to follow the airstream; thus, they have a better chance at exiting the sensor. Still, if one is concerned by the effect of small particles, the CSR’s performance can be further improved by operating the CSR at higher airflow rates. This approach is effective since increasing the airstream velocity also increases the centrifugal power affecting the dust particles. As these forces increase, smaller particles are removed from the airstream. This approach is certainly acceptable, but it increases the energy consumption of the CSR. Inoya et al. developed an alternative method [26] which resulted in a considerable improvement in CSRs’ capability to remove fine dust particles without increasing energy consumption. In this version, there are two airstreams, the main one from the CSR inlet and another from the dustbin (see left part of Figure 1). This modification significantly improves the CSR’s capability to protect the sensor at extreme conditions [8] while maintaining the total airflow rate. Figure 2 shows the dust removal efficiency of the conventional and improved CSR using the same total airflow in both cases as a function of particle size. It shows that, in both configurations, dust particles with a diameter equal or greater than 4 micrometers are removed from the airstream, and the removal efficiency is very close to 100% for these particles. For smaller particles, the effectiveness is decreased in both cases. However, using improved CSR is significantly more effective in removing small particles. For example, the CSR removal efficiency for 0.3 micrometers is ~10% by the CSR and ~45% by the improved CSR.

## 3. Conclusions

The immense technological development in the modern age has many positive outcomes for humans but often harms the environment. Reducing the environmental impact of this development should be our primary goal today for a better future for generations to come. One of the areas that we should focus our efforts on is air monitoring. Detailed information regarding pollution levels is crucial to focus the mitigating and regulating actions. In recent years, sensing technologies have evolved considerably. Sensors are small, affordable, and automatic; communicating the information to the decision-makers is easier than ever. Sensors have to become more rugged by adopting technologies that are common in harsh industrial sites; thus, they can withstand sub-optimal operational conditions that are common in outdoor operation. However, protecting gas sensors from aerosols is based on particulate filters, which require frequent maintenance and degrade the sensor’s performance. Studies show that climate change in the last century has increased aerosol concentration, making prolonged gas sensing more challenging than ever.

The success in protecting CWA sensors from high dust concentrations at battlefield conditions using the CSR suggests that this technology can be adapted for air sensing in urban areas. The CSR provides an adequate solution as it is practically a maintenance-free, effective add-on unit that can be coupled to most commercially available sensors without modifying them. Such an application of the CSR should be made after careful consideration of the application characteristics such as the dust concentration that the sensor will face, the sensor’s vulnerability to dust, and the target material’s characteristics. If the simple solution of using a particulate filter is impractical due to high maintenance requirements and possible adsorption of organic vapor (such as aromatic hydrocarbon), the CSR can be a useful and practical alternative.
